# Editorial: New Genome Editing Tools and Resources: Enabling Gene Discovery and Functional Genomics

**DOI:** 10.3389/fgeed.2021.771622

**Published:** 2021-10-08

**Authors:** Wendy Harwood, Qi-Jun Chen, Feng Zhang, Bing Yang

**Affiliations:** ^1^ John Innes Centre, Norwich Research Park, Norwich, United Kingdom; ^2^ State Key Laboratory of Plant Physiology and Biochemistry, College of Biological Sciences, China Agricultural University, Beijing, China; ^3^ Department of Plant and Microbial Biology, University of Minnesota, St. Paul, MN, United States; ^4^ Center for Precision Plant Genomics, University of Minnesota, St. Paul, MN, United States; ^5^ Center for Genome Engineering, University of Minnesota, St. Paul, MN, United States; ^6^ Division of Plant Sciences, Bond Life Sciences Center, University of Missouri, Columbia, MO, United States

**Keywords:** genome editing, gene discovery, functional genomics, CRiSPR/Cas, crop improvement

Genome editing technologies are revolutionizing molecular biology research and offer huge potential for development of crops that could help meet the challenge of providing sufficient food, sustainably and under increasingly challenging environmental conditions. The ability to make precise changes in plant genomes, together with the increased genomic resources now available, give unprecedented opportunities to develop crops with desired traits much faster than with traditional techniques. Although there are a range of genome editing technologies available, the one that is currently most widely used, and has generated the most excitement, is CRISPR/Cas. Despite the great progress of CRISPR/Cas-induced genome editing in plants, two main challenges persist: delivery of CRISPR reagents and precise genome editing. The papers in this research topic all feature CRISPR-based systems and highlight some of the latest advances in this fast-moving area including in delivery and precise genome editing technologies.

**FIGURE 1:**
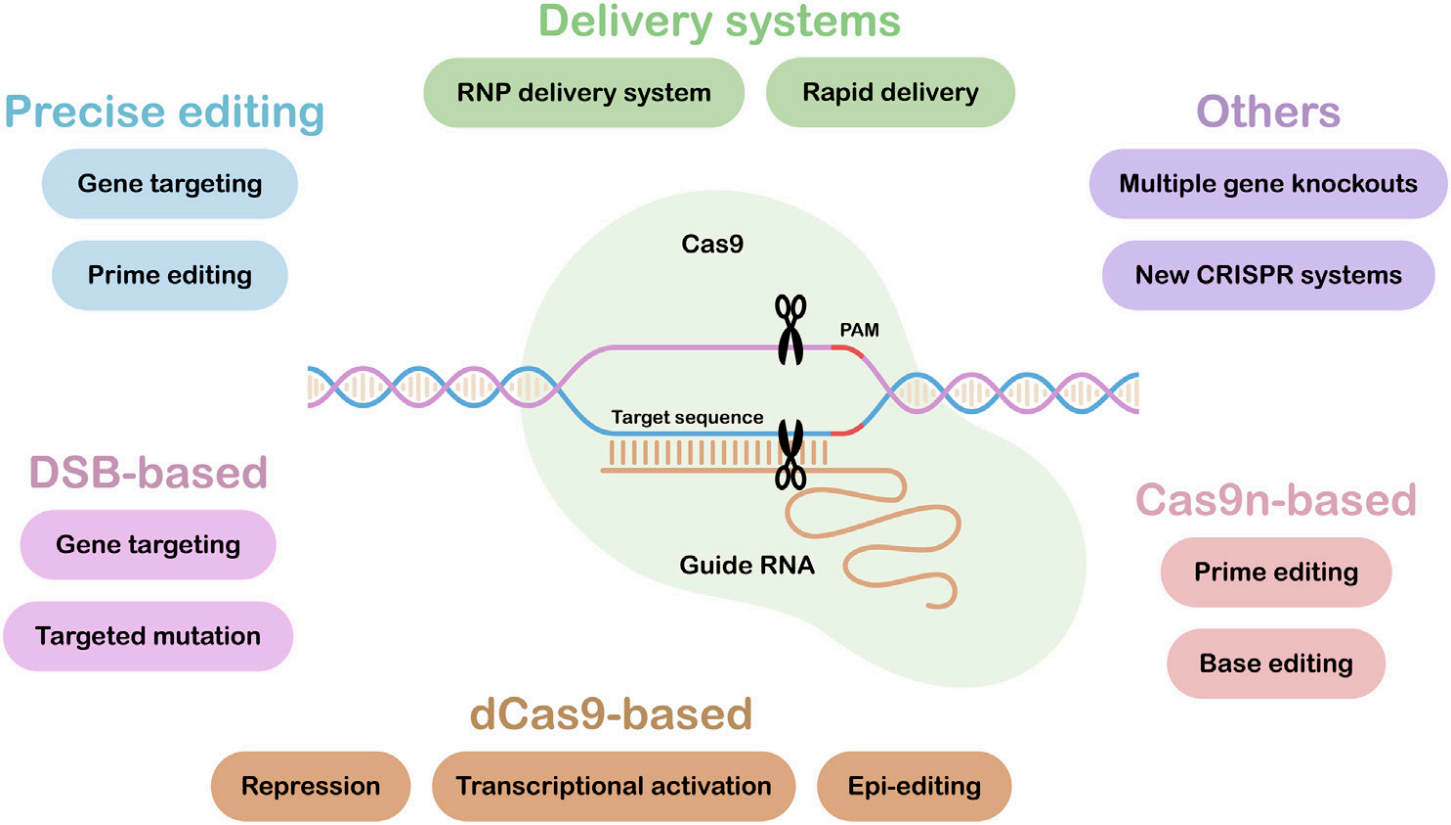
CRISPR/Cas Tools and Resources. Tools and Resources under the headings: Precise editing, Delivery systems and Others are covered in this research topic. The lower part of the figure summarizes current Cas9-based tools and applications. DSB, Double Strand Break; dCas9, dead or inactive Cas9; Cas9n, Cas9 nickase.

CRISPR/Cas applications have rapidly moved from allowing simple, single target gene knock-outs to enabling more complex targeted edits. Figure 1 illustrates the range of tools and resources now available, with those under the headings: Precise editing, Delivery systems and Others highlighted in this research topic. Some of our most important crops have polyploid genomes with multiple gene copies. This can complicate editing strategies (Schaart et al., 2021), however, in the paper entitled “*Multiallelic, Targeted Mutagenesis of Magnesium Chelatase With CRISPR/Cas9 Provides a Rapidly Scorable Phenotype in Highly Polyploid Sugarcane*” Eid et al. show that up to 49 out of 59 copies of the target gene, magnesium chelatase, could be mutated using just two sgRNAs. It was also shown that a heat treatment could increase editing efficiencies 2-fold, while also promoting editing of multiple copies of the target gene.

One attraction of genome editing is that once the required edits have been achieved, the editing components integrated into the genome can be segregated away in subsequent generations, leaving a plant with the required edit only and no foreign DNA. An alternative to this approach is to introduce the editing components as a ribonucleoprotein (RNP) complex (Zhang et al., 2021). Dong et al. in their manuscript “*Efficient Targeted Mutagenesis Mediated by CRISPR-Cas12a Ribonucleoprotein Complexes in Maize*,” use this RNP approach, but rather than the common Cas9 RNP, they use a Cas12a RNP and deliver this into maize protoplasts and immature embryos. This RNP approach gave average editing efficiencies of over 60%; comparable to or higher than efficiencies achieved by editing components from transgenes. Several versions of Cas12a have been reported and a comparison by the authors showed improved editing with some Cas12a variants.

Another enhanced Cas12a (FnCas12a) is reported by Negishi et al. in their paper *“Enhanced FnCas12a-Mediated Targeted Mutagenesis Using crRNA with Altered Target Length in Rice*.” The authors report that the efficiency of FnCas12a-mediated editing depends on the length of the crRNA guide sequence. Altering the length of the crRNA changed the frequency with which large deletions could be obtained, indicating that this approach could fine-tune the editing outcome. The two papers describing the use of Cas12a add to the current literature demonstrating the high potential and versatility of this nuclease family (Bandyopadhyay et al., 2020).

In addition to single and multiple targeted gene knock-outs, there is a need to make other, specific targeted changes in plant genomes. Where a single base change is required, then base editing approaches may be appropriate (Figure 1). However, where more than one base change is required, the new technique of prime editing may be used. In the paper “*Spelling Changes and Fluorescent Tagging with Prime Editing Vectors for Plants*,” Wang et al. describe a set of easy-to-use vectors for prime editing in both dicot and monocot species. Generally, the size of insertion achieved by prime editing is small (Lin et al., 2020), but Wang et al. showed in their paper that it is possible to insert 66 bp, the largest reported to date. To make even larger genomic insertions at a precise location, gene targeting is required. The targeted insertion of large sequences or entire genes is technically challenging, and efficiencies are generally low (Dong and Ronald 2021). In the paper by Lawrenson et al. “*In-planta Gene Targeting in Barley using Cas9 with and without Geminiviral Replicons*,” successful gene targeting in barley is described, with an mCherry marker gene being inserted at the target genomic locus.

As well as tools that expand the range of possible genome editing applications, ways to improve the speed and efficiency of genome editing systems have been examined. The process of plant genome editing can be time consuming, as there is generally a need for regeneration of plants from tissue culture. It is important, therefore, to have confidence that specific genome editing components will work. Nasti et al. in their paper “*Fast-TrACC: A Rapid Method for Delivering and Testing Gene Editing Reagents in Somatic Plant Cells*” address this issue. They describe a system that uses a luciferase reporter to provide a readout of the efficiency of *Agrobacterium*-mediated delivery of genome editing reagents. The ability to test sgRNAs before attempting plant genome editing can save valuable time.

Often it is the generation time of a crop plant that limits fast progress in genome editing. In certain crops, rapid flowering lines have been developed. Fast-flowering mini-maize is one such example (McCaw et al., 2016). As well as the fast-flowering phenotype, mini-maize also needs to be amenable to transformation to make it valuable for rapid genome editing applications. In the paper by McCaw et al. in this research topic, “*Development of a Transformable Fast-Flowering Mini-Maize as a tool for Maize Gene Editing*,” the authors describe development of a fast-flowering mini-maize that is also amenable to transformation and editing at efficiencies up to 17 and 79%, respectively, with a seed to T1 seed time of 5.5 months compared to over 9 months for other genotypes.

In summary, this collection of papers highlights some exciting recent developments in provision of CRISPR/Cas tools and resources. These enhanced resources are poised to make a major contribution to more efficient and rapid gene discovery and functional characterization.
